# Evaluation of the clinical and quantitative performance of a practical HPLC-UV platform for in-hospital routine therapeutic drug monitoring of multiple drugs

**DOI:** 10.1186/s40780-023-00298-7

**Published:** 2023-10-01

**Authors:** Go Morikawa, Kazuto Fukami, Yukiko Moriiwa, Katsuko Okazawa, Akio Yanagida

**Affiliations:** 1 Department of Pharmacy, Hokushin General Hospital, 1-5-63, Nishi, Nakano, Nagano, 383-8505 Japan; 2 Department of Clinical laboratory, Hokushin General Hospital, 1-5-63, Nishi, Nakano, Nagano, 383-8505 Japan; 3 Department of Biomedical Analysis, School of Pharmacy, Tokyo University of Pharmacy and Life Sciences, 1432-1 Horinouchi, Hachioji, Tokyo, 192-0392 Japan

**Keywords:** Therapeutic drug monitoring, HPLC-UV, Solid-phase extraction, Anticonvulsant, Antimicrobial

## Abstract

**Background:**

In-hospital therapeutic drug monitoring (TDM) requires a suitable quantification method for target drugs from the viewpoint of precision, throughput, and testing costs. We previously developed a practical HPLC-UV platform for quantification of serum levels of various drugs. In this report, the platform was effectively applied to the quantification of patient serum levels of five different drugs by clinical professionals in our hospital during their daily work.

**Methods:**

The residual sera of patients receiving carbamazepine (CBZ), phenytoin (PHT), lamotrigine (LTG), vancomycin (VCM), or voriconazole (VRCZ) were used in the present clinical study. The quantification method for each drug consisted of rapid solid-phase extraction (SPE) of each drug in the patient serum, followed by optimized HPLC-UV analysis of the drug in the SPE eluate. Furthermore, patient serum levels of PHT, CBZ, and VCM were also measured by ligand-binding assay using a cobas^®^ analyzer in our hospital, and those of LTG and VRCZ were measured by HPLC-MS/MS at an outsourced provider. Passing–Bablok regression analysis and Bland–Altman analysis were employed to analyze the agreement of drug levels in patient sera, which was separately quantified using two different methods—our HPLC-UV platform and the cobas analyzer, or HPLC-UV and HPLC-MS/MS.

**Results:**

All analytical conditions of the present method using our HPLC-UV platform were well optimized for each target drug quantification in the patient’s serum, and the quantification method for each drug was fully validated for accuracy, precision and reproducibility. Furthermore, Passing–Bablok regression analysis and Bland–Altman analysis revealed that patient serum levels of PHT, CBZ, and VCM quantified by our HPLC-UV platform were closely correlated with those quantified by the cobas^®^ analyzer, and the levels of LTG and VRCZ quantified by our HPLC-UV platform were also correlated with those quantified by HPLC-MS/MS.

**Conclusions:**

Our HPLC-UV platform can be performed without requiring special analytical techniques. This platform is expected to be used for the measurement of blood levels of multiple drugs for in-hospital routine TDM.

**Supplementary Information:**

The online version contains supplementary material available at 10.1186/s40780-023-00298-7.

## Background

The implementation of therapeutic drug monitoring (TDM), based on the quantification of blood drug levels, is crucial for assessing the efficacy and safety of an administered drug. Thus, TDM analysts must have adequate quantification methods for the target drugs from the viewpoint of precision, throughput and testing costs. Generally, ligand-binding assay (LBA) and high-performance liquid chromatography (HPLC) have been used as analytical methods for the quantifications of blood levels of numerous kinds of drugs [1, 2]. LBA, particularly immunoassay using specific antibodies, is a suitable method for the automation of drug analysis for TDM, and thus several types of high-speed clinical LBA analyzers currently operate in most general hospitals in Japan. However, as most LBA methods for TDM indirectly quantify the blood level of a target drug by binding with its antibody and subsequent signal amplification, they suffer from low quantitative capability due to antibody cross-reactivity [1]. Notably, they are not applicable to the analysis of drugs that lack corresponding antibodies. Meanwhile, most HPLC methods for TDM directly quantify the blood levels of target drugs using online detectors after chromatographic separation. Thus, the quantitative capability (in terms of specificity, accuracy, repeatability, and precision) of HPLC methods is higher than that of LBA methods [2].

Among all HPLC platforms for TDM, HPLC with tandem mass spectrometry (HPLC-MS/MS) shows superior performance in terms of sensitivity, selectivity, and universality [1]. Moreover, reports of the application of HPLC-MS/MS in clinical laboratories have been increasing recently [3–6]. However, as the installation of an HPLC-MS/MS apparatus entails high costs, owing to the operation/maintenance of the complicated apparatus, HPLC-MS/MS methods have not been widely used for routine TDM analysis in general hospitals in Japan. Thus, hospitals with neither LBA analyzers nor HPLC-MS/MS have no choice but to depend on time-consuming outsourced services for TDM analysis [7].

Given the discussed limitations of HPLC-MS/MS, the practical value of a conventional HPLC apparatus with ultraviolet (UV) absorption detection has been once again recognized and has drawn attention for routine TDM analysis in clinical practice. For example, Tuchishita et al. reported the clinical and economic practicality of TDM of several antiarrhythmic agents through in-hospital routine quantitation using a conventional HPLC-UV apparatus [8]. Other researchers also reported the usefulness of a simple economic HPLC-UV method for TDM focusing on several antibacterial agents [9, 10]. Meanwhile, the HPLC conditions described in the above reports (e.g., pre-extraction procedure, type and size of the separation column, flow rate and composition of the mobile-phase solvent, and detection UV wavelength) were different for each drug and were not unified as a single HPLC platform for multiple drug analyses. Thus, in each hospital, resetting the HPLC conditions for each target drug on a case-by-case basis is difficult and represents a burden for medical workers.

Given these circumstances, we previously developed a practical HPLC platform for the in-hospital quantification of serum levels of various drugs [11]. The platform consists of the simple solid-phase extraction (SPE) of a drug in serum using a disposable centrifugal cartridge, followed by rapid HPLC quantification of the drug in the SPE eluate using an easy-to-use reversed-phase (RP) HPLC-UV apparatus. We further demonstrated that the platform could be applied for the quantification of the serum levels of 15 different drugs (carbamazepine [CBZ], phenytoin [PHT], lamotrigine [LTG], disopyramide, flecainide, lidocaine, mexiletine, procainamide, propafenone, quinidine, sotalol, voriconazole [VRCZ], mycophenolic acid, imatinib, and pazopanib) with almost the same procedures and exactly the same HPLC-UV apparatus. Furthermore, the method was also applied to the clinical evaluation of the blood levels of favipiravir [12, 13] and VRCZ [14] at different hospitals in Japan. However, with a few exceptions such as favipiravir and VRCZ, the method using the above platform is just beginning to be applied to the clinical evaluation of blood levels of other kinds of drugs during therapy in clinical practice.

In this report, our practical HPLC-UV platform was effectively applied to the quantification of serum levels of five different drugs (CBZ, PHT, LTG, VRCZ and vancomycin [VCM]) in our hospital by clinical laboratory technicians and pharmacists during their daily work. Furthermore, the serum levels of each drug quantified by our platform were compared with those quantified by an LBA analyzer and/or HPLC-MS/MS analysis to confirm the quantitative capability of our practical method.

## Methods

### Chemical reagents and drugs

PHT (purity: >99%), LTG (purity: >99%), VCM (purity: >93.5%), and ammonium acetate trihydrate were purchased from FUJIFILM Wako Pure Chemical (Osaka, Japan). CBZ (purity: >97%) and VRCZ (purity: >98%) were purchased from Tokyo Chemical Industry (Tokyo, Japan). Acetonitrile (CH_3_CN; HPLC grade) was purchased from Kanto Chemical Co., Inc. (Tokyo, Japan). Sterile purified water was purchased from Hikari Pharmaceutical Co. (Tokyo, Japan). PHT, LTG, VCM, CBZ, and VRCZ were used as standard compounds for the quantification of each drug content in patient sera (described in the following section). Meanwhile, normal human serum (NHS) was purchased from FUJIFILM Wako Pure Chemical for the validation of the present quantification method.

### Patient sera

Residual patient sera recovered after a general blood test for medical treatment were used in this study. The sera were obtained from patients treated with CBZ, PHT, LTG, VCM, or VRCZ at Hokushin General Hospital. The protocol of the present study was approved by the Ethics Committee of the hospital (Receipt No. 2,016,004), and all experiments using patient residual sera were performed at the hospital under blinded conditions using each patient’s ID. Since patient serum was used in this study for the purpose of evaluating this HPLC-UV platform, the results of the measurements were not used for clinical use.

### SPE cartridge and optimized SPE protocol

**Fig. 1:**
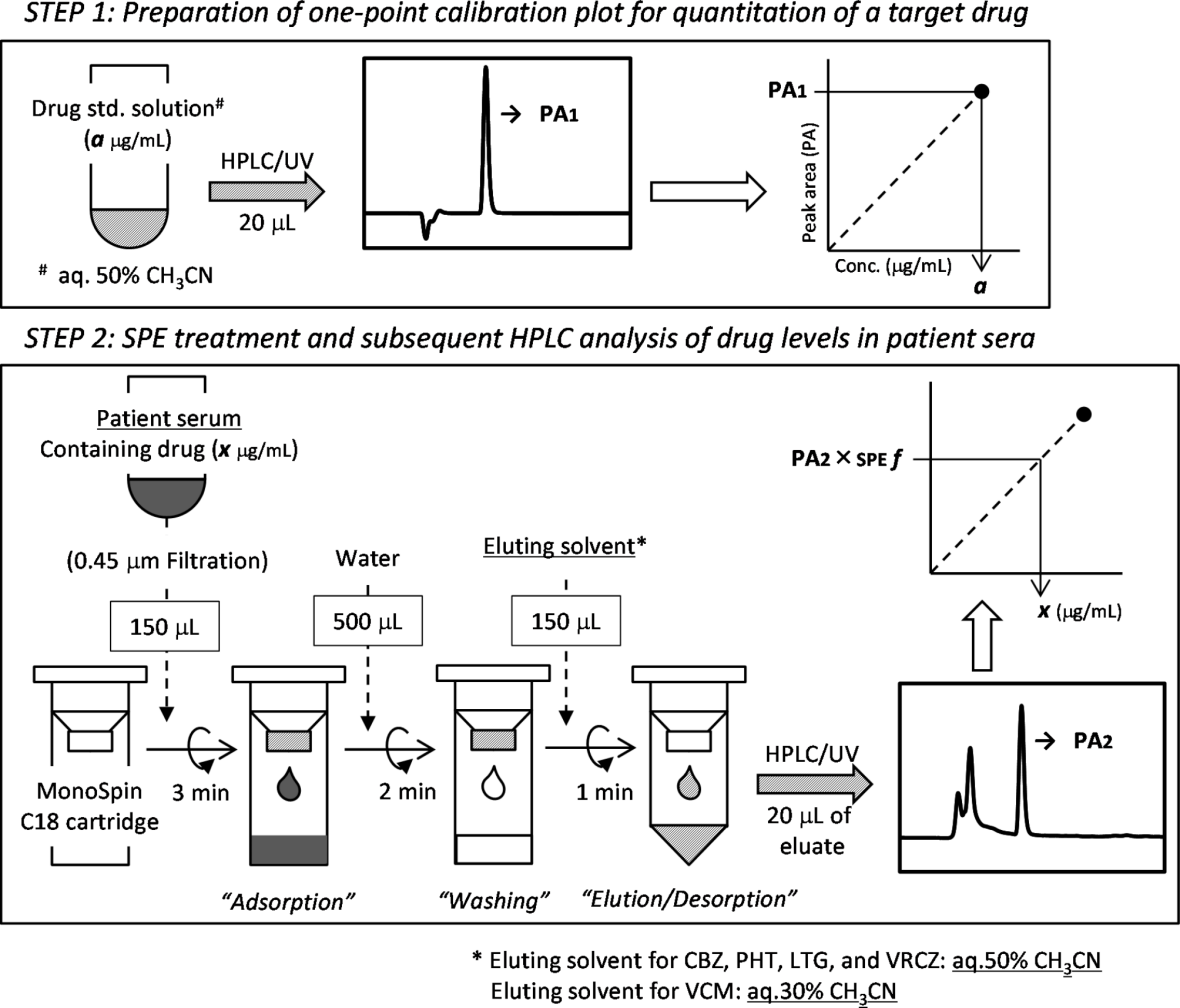
Schematic of the present quantification method of drug levels in patient sera

A monolithic C_18_-silica disk built-in centrifugal spin-cartridge, MonoSpin C_18_ (GL Sciences, Inc., Tokyo, Japan), was chosen as an easy-to-use tool for SPE treatment before the HPLC analysis of each drug level in the patients’ sera. The MonoSpin cartridge was pretreated through the sequential passage of 500 µL each of CH_3_CN and water before use. Each solution was passed through the cartridge by centrifugation at 5,000 rpm (2,400×g) for 1 min using a centrifuge (Himac CT15E; Koki Holdings, Tokyo, Japan). Details of the SPE procedure using the pretreated cartridge are as follows. First, the patient sera were filtered using a DISMIC 13HP syringe filter (0.45 μm; ADVANTEC, Tokyo, Japan), and each filtered serum (150 µL) was loaded onto a MonoSpin C_18_ cartridge through centrifugation for 3 min. Second, water (500 µL; wash solution) was passed through the cartridge through centrifugation for 2 min. Third, an aqueous solution containing CH_3_CN (150 µL; eluting solution) was passed through the cartridge through centrifugation for 1 min, and the final eluate containing the drug was collected in a test tube for subsequent HPLC analysis. The optimized protocol of the SPE method for quantifying the serum level of each drug (CBZ, PHT, VCM, LTG, or VRCZ) is shown schematically in Fig. 1. Eluting solvent for CBZ, PHT, LTG, and VRCZ was aq.50% CH_3_CN, while eluting solvent for VCM was aq.30% CH_3_CN.

### HPLC apparatus and optimized HPLC conditions

All HPLC analyses were conducted using a Chromaster system (Hitachi High-Tech Science Corporation, Tokyo, Japan). The system consisted of a 5310 column oven, a 5210 autosampler, a 5110 pump, and a 5430 diode-array detector. The RP-HPLC separation of each drug (PHT, LTG, VCM, CBZ, or VRCZ) was performed at 40 °C (default temperature) on a Chromolith HighResolution RP-18 column (100 mm × 4.6 mm i.d.; Merck, Darmstadt, Germany) equipped with a guard column (5 mm × 4.6 mm i.d.). All HPLC analyses were performed in isocratic or gradient elution mode using a mixture of two mobile-phase solvents: A (CH_3_CN) and B (10 mM acetate buffer, pH 5.0). For example, an analyte containing CBZ (injection volume: 20 µL) was simply eluted with a solvent mixture (A:B = 40:60, v/v) at a flow rate of 2.0 mL/min over 3 min, and the CBZ in the eluate was detected using UV absorption at 280 nm. Details of the optimized HPLC conditions of all five drugs are listed in Tables S1 and S2 in the Supplementary data.

### Validation method

A one-point calibration curve for the HPLC quantitation of the serum level of each drug was prepared from HPLC data of the SPE eluate of serum spiked with each drug (for CBZ, PHT, or LTG: 20 µg/mL [in NHS]; for VCM: 50 µg/mL; for VRCZ: 5 µg/mL) without the use of an internal standard (IS) compound. The limit of detection (LOD), the limit of quantitation (LOQ), and the lower limit of quantitation (LLOQ) of each drug were estimated from a chromatogram of the SPE eluate of serum spiked with each drug (for CBZ, PHT, or LTG: 2 µg/mL [in NHS]; for VCM: 3 µg/mL; for VRCZ: 1 µg/mL) at the signal-to-noise ratios of 3:1, 10:1, and 5:1, respectively.

An intraday study (repeatability) of the present quantitation method was performed by analyzing the quality control (QC) samples (e.g., for PHT, these were the sera spiked with PHT at 2, 10, and 20 µg/mL) four times during the same day, whereas an interday study (intermediate precision) was performed by analyzing the QC samples once-per-day on separate days. The accuracy was reported by calculating the bias, expressed as (measured concentration)/(normal concentration) × 100%; the precision was reported as the coefficient of variation, expressed as (SD/mean of measured concentration) × 100%.

The repeatability (at three times) of the quantitation of a drug level in the identical patient serum was also examined by the identical analyst (clinical technologist) in our hospital. The numbers of patient sera for this examination were 13 for CBZ, four for PHT, two for LTG, and two for VRCZ.

### Quantitation of drug level in patient serum using the “cobas” LBA analyzer

The patient serum levels of CBZ, PHT, and VCM were also measured by the LBA method using a fully automated cobas^®^ 6000 < 501 > analyzer (Roche Diagnostics, Tokyo, Japan) with electrochemiluminescence technology. The numbers of patient sera for this measurement were 23 for CBZ, 20 for PHT, and 20 for VCM. The cobas analyzer is routinely used for the measurement of blood levels of seven kinds of drugs (CBZ, PHT, VCM, phenobarbital, sodium valproate, digoxin, and theophylline) in our hospital. In this study, all quantitative measurements using the cobas analyzer were performed as a routine part of the clinical laboratory technician’s job.

### Quantitation of drug level in patient serum using HPLC-MS/MS

The patient serum levels of LTG and VRCZ were also measured using HPLC-MS/MS at the outsourced provider (SRL, Inc., Tokyo, Japan). The number of patient sera for this measurement was nine each for LTG and VRCZ. SRL routinely offers clinical laboratory testing, receiving consigned specimens from hospitals, and has proven quality assurance systems and reporting on test results. In this study, details of the measurement conditions of HPLC-MS/MS were not disclosed to us by SRL. It can take up to 1 week to obtain feedback on the quantitative result from SRL after sending in the patient’s serum.

**Data plotting methods used in analyzing the agreement between two different quantification methods for serum drug level**.

Passing–Bablok regression analysis, Bland–Altman analysis, and Pearson’s correlation coefficient were employed to analyze the agreement of drug levels in patient sera, which was separately quantified using two different methods—our HPLC-UV platform and the cobas analyzer, or HPLC-UV and HPLC-MS/MS. A Bland–Altman plot [15] of a total of 75 data quantified by HPLC-UV and another method (cobas or HPLC-MS/MS) was constructed between the mean of the drug levels quantified by both methods and the difference between these drug levels. Bias was estimated as the mean of the differences between both methods. The upper and lower limits of agreement were plotted with a 95% confidence interval (as the limit of agreement [LOA] = mean ± 1.96 × SD). The analyses were performed using Microsoft^®^ Excel^®^ for Microsoft 365 MSO (version 2303 build 16.0.16227.20202) 64-bit.

## Results

### HPLC-UV profiles of the target drugs after method optimization

In advance of the present clinical study, our previous methods [11] for the quantification of serum levels of four different drugs (CBZ, PHT, LTG, or VRCZ) were slightly modified and optimized to the patient sera in our hospital. Specifically, all patient sera were passed through a syringe filter (0.45 μm) before SPE treatment, and centrifugation times of the SPE cartridge were re-adjusted for each step. The schematic procedure of the present drug quantification method in patient sera is shown in Fig. 1. The working time for the SPE treatment series for each drug was within 10 min, and a clinical laboratory technician or pharmacist was able to carry out SPE treatment of the patient sera between routine tasks in our hospital.

Furthermore, we additionally planned to determine the VCM level in patient sera by our quantification method, and the analytical conditions of the method were newly optimized for VCM quantification. Details of the well-optimized conditions of the SPE treatment for VCM are also shown in Fig. 1, and those of the HPLC-UV operation for VCM are found in Tables S1 and S2.

**Fig. 2:**
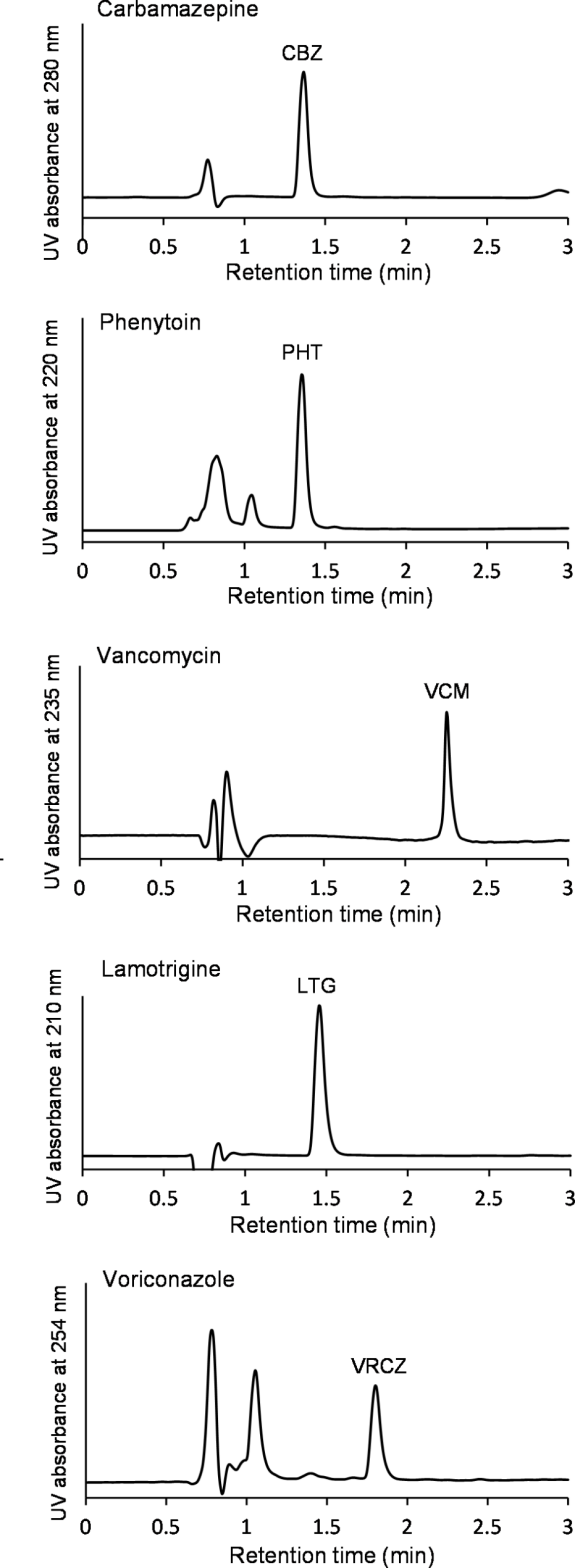
HPLC-UV chromatograms of carbamazepine (CBZ), phenytoin (PHT), vancomycin (VCM), lamotrigine (LTG) and voriconazole (VRCZ) detected from patient sera by the present quantification method

After the above method optimization, each optimized method for CBZ, PHT, VCM, LTG, or VRCZ was respectively applied to quantify each drug level in a patient’s serum. Figure 2 shows the HPLC-UV chromatograms of the five target drugs detected from patient sera. In all chromatograms, each drug clearly appeared as a sharp single peak without any interrupted signals, and was rapidly eluted within three minutes.

### Validation of the present quantification method using the HPLC-UV platform

Additionally, the analytical performance of the present quantification method for five drugs using the HPLC-UV platform was validated by the use of serum spiked with each drug, and the validation results are shown in Tables S3, S4 and S5. Table S3 shows the recovery efficiency (recovery rate and SPE factor) and Table S4 shows limit values (LOD, LOQ, and LLOQ) of each drug. The recovery rates were appropriate, between 87.9% (for PHT) and 108.5% (for VRCZ), and all LLOQ values were less than 1 µg/mL. Table S5 shows the accuracy and precision data of the present quantification method for each drug. The accuracy data showed suitable values between 91.3% (for 50 µg/mL of VCM) and 108.3% (for 2 µg/mL of CBZ). Further, all data of repeatability and intermediate precision showed excellent values of less than 10%, with the exception of those for 1 µg/mL of VCM (10.6% and 18.6%, respectively).

Meanwhile, the repeatability (at three times) of the quantitation of a drug level in the identical patient serum was also examined by the identical analyst (clinical technologist). The number of patient sera for this examination was 13 for CBZ, four for PHT, two for LTG, and two for VRCZ (total *n* = 21). The relative standard deviation (RSD) value of each measurement ranged from 1.1 to 16.7%, indicating that the repeatability was highly reliable, as shown in Table S6.

### Evaluation of the agreement between two different methods (HPLC-UV and cobas, or HPLC-UV and HPLC-MS/MS)

**Fig. 3:**
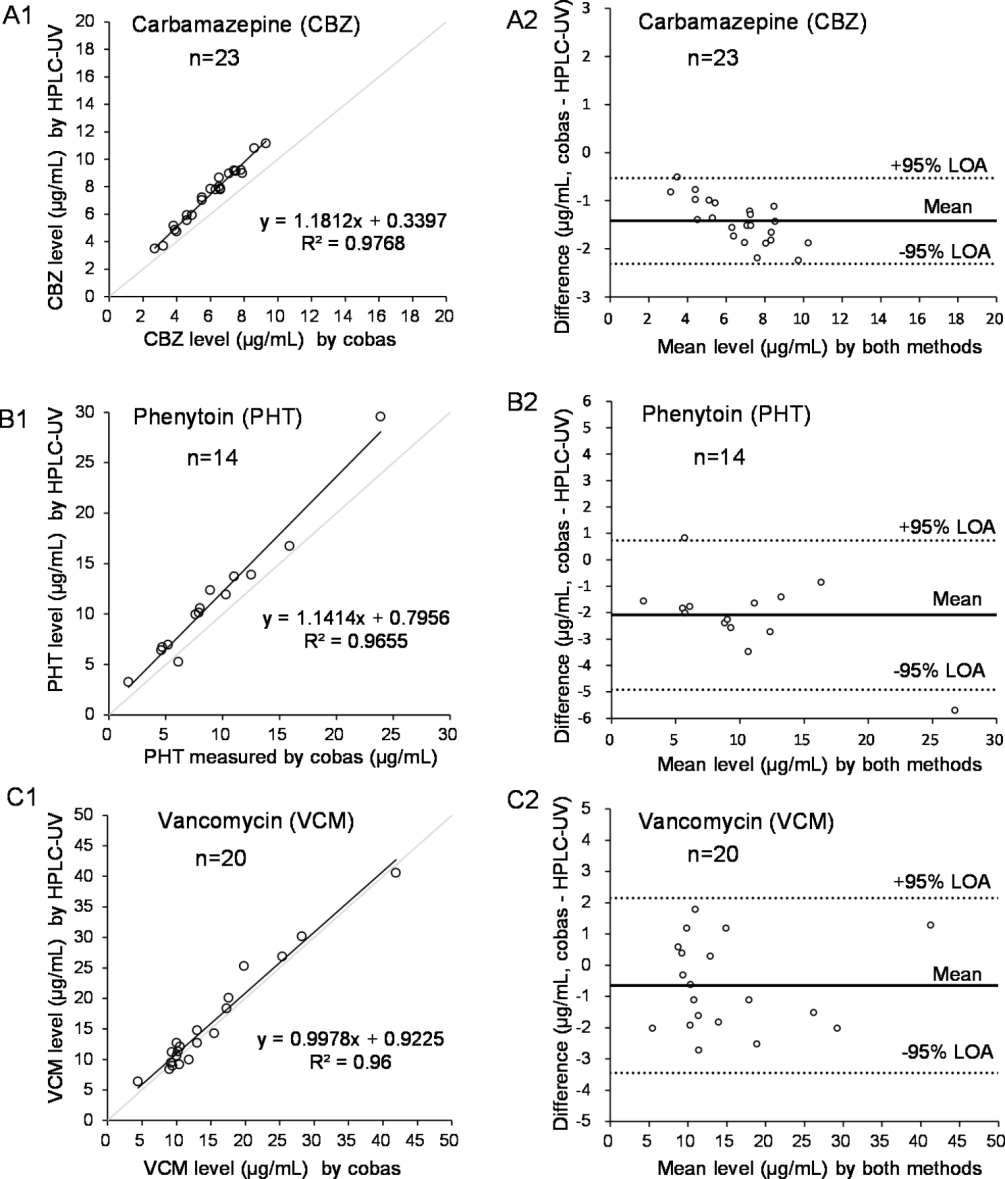
Comparison of Passing–Bablok regression analysis (A1, B1, C1) and Bland–Altman analysis (A2, B2, C2) results of serum levels of the three drugs (CBZ, PHT, VCM), which were separately quantified by the present HPLC-UV platform and by the cobas^®^ analyzer. Both quantifications were performed in our hospital

Figure 3 shows the results of Passing–Bablok regression analysis and Bland–Altman analysis of the serum drug levels (CBZ, PHT, and VCM), which were separately quantified by the present HPLC-UV platform and a cobas LBA analyzer in our hospital. As shown in the Passing–Bablok regression plots A1, B1, and C1, the regression coefficient between the CBZ levels by HPLC-UV and by cobas was 1.1812 (*n* = 23, coefficient of determination [*R*^2^] = 0.9768), that between the PHT levels by both methods was 1.1414 (*n* = 14, *R*^2^ = 0.9655), and that between the VCM levels by both methods was 0.9978 (*n* = 20, *R*^2^ = 0.9600). The cobas assay for CBZ and PHT showed a substantial difference of − 1 to − 2 µg/mL compared with our HPLC method. Furthermore, among the Bland–Altman plots (between HPLC-UV and cobas) shown in A2, B2, and C2, the highest mean difference was observed from the PHT data in B2 (–2.1 ± 1.4 µg/mL). In the Bland–Altman plots, the 95% LOA data ranged from − 2.3 to − 0.5 µg/mL (CBZ in A2), − 4.9 to 0.7 µg/mL (PHT in B2), and − 3.4 to 2.1 µg/mL (VCM in C2).

**Fig. 4:**
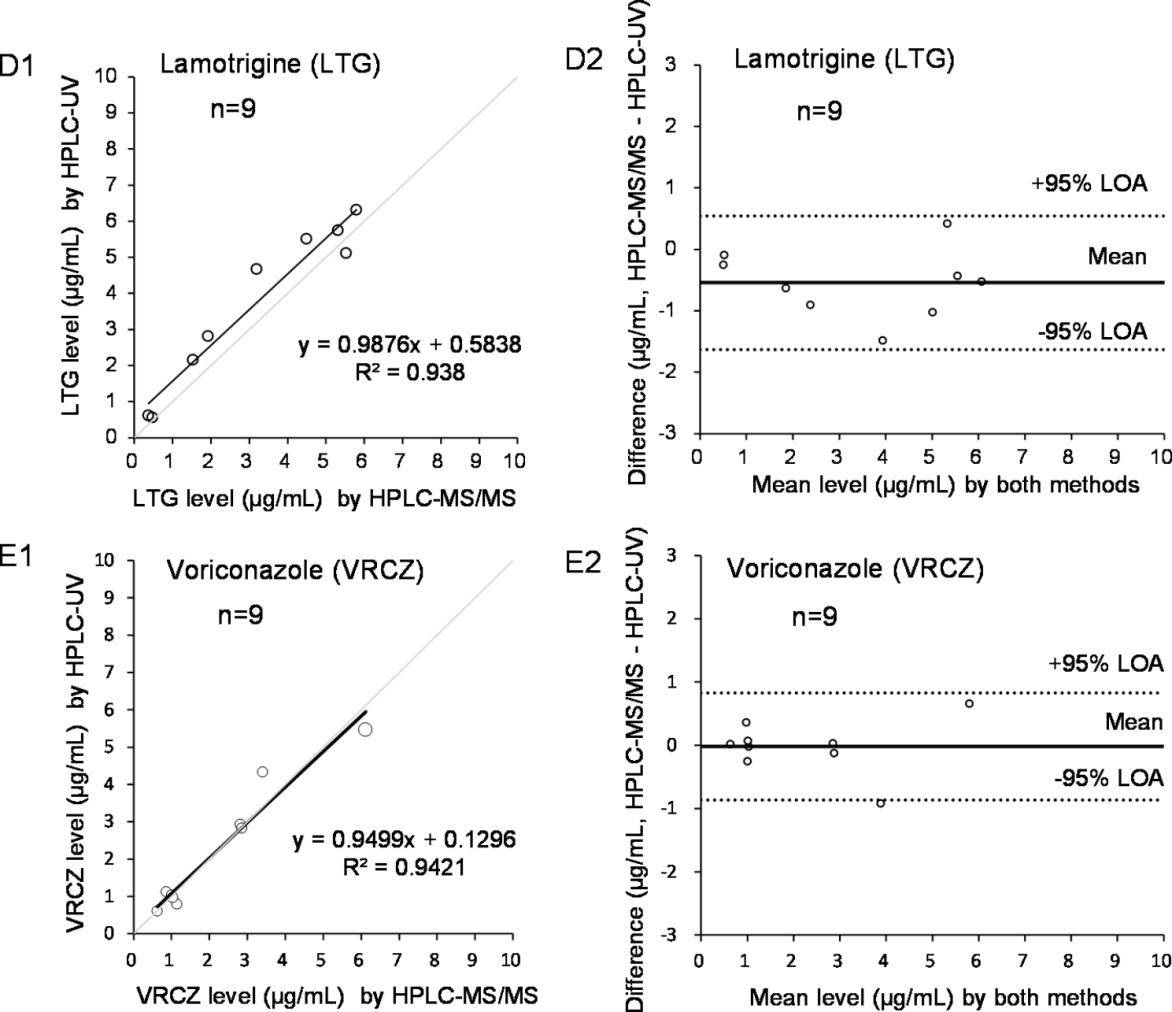
Comparison of Passing–Bablok regression analysis (D1, E1) and Bland–Altman analysis (D2, E2) results of the serum levels of two drugs (LTG, VRCZ), which were separately quantified by the present HPLC-UV platform and by a HPLC-MS/MS system. Quantification by HPLC-UV was performed in our hospital, while that by HPLC-MS/MS was conducted by the outsourced provider (SRL, Inc.)

Furthermore, Fig. 4 shows the results of Passing–Bablok regression analysis and Bland–Altman analysis of serum drug levels (LTG and VRCZ), which were separately quantified by the present HPLC-UV platform in our hospital and by the outsourced HPLC-MS/MS analysis. As shown in the Passing–Bablok regression plots D1 and E1, the regression coefficient between the LTG levels by HPLC-UV and by HPLC-MS/MS was 0.9876 (*n* = 9, *R*^*2*^ = 0.9380), and that between the VRCZ levels by both methods was 0.9499 (*n* = 9, *R*^*2*^ = 0.9421), respectively. Furthermore, among the Bland–Altman plots (between HPLC-UV and HPLC-MS/MS) shown in D2 and E2, the 95% LOA data ranged from − 1.6 to − 0.6 µg/mL (LTG in D2) and − 0.9 to 0.8 µg/mL (VRCZ in E2), respectively.

## Discussion

The HPLC-UV platform used in this study was constructed with an emphasis on practicality and economy, enabling its use in many hospitals. In addition, the operator was assumed to be a staff member unfamiliar with HPLC analysis. Although the analysts who performed the measurements in this study were unfamiliar with HPLC analysis, the results of the repeatability of drug quantitation in the same patient serum samples were reliable and reproducible (as shown in Table S6), indicating that the platform’s procedure is straightforward (Fig. 1). Furthermore, the validation results of the present quantification method using our HPLC-UV platform were suitable (Tables S4 and S5), indicating acceptable performance for handling daily TDM operations. However, separate confirmation is required to assess the accuracy of quantification of concentrations above the therapeutic range.

The quantitative values of the HPLC-UV method correlated well with those of the other methods (Figs. 3 and 4); however, the values of the HPLC-UV method for CBZ and PHT were higher than those of the cobas assay (Fig. 3). This is because the HPLC method directly quantifies blood levels of the target drugs using online detectors after chromatographic separation, thus, the quantitative capability of HPLC methods is higher than that of LBA methods [2]. Since the accuracy of CBZ and PHT using this HPLC-UV platform is close to 100% (as shown in Table S5), it is concluded that the results using the cobas are slightly lower and we should be used with caution when performing TDM. In addition, as shown in Fig. 4 for VRCZ, the 95% LOA range of determination of VRCZ by this platform was found to be close to ± 1 µg/mL, and the actual difference of 1 µg/mL of serum VRCZ between other analytical methods will be critical in patients because the range of safety levels of serum VRCZ is known to be relatively low and narrow. Because of the small sample size in this study, it is worthwhile to further investigate its impact.

In this HPLC-UV platform, we confirmed the feasibility of clinical application through the optimization of (1) pretreatment conditions, (2) HPLC/UV conditions, and (3) the absolute calibration curve method.

First, for the pretreatment conditions, MonoSpin (a centrifugal spin cartridge) was selected as the disposable solid-phase cartridge for SPE. The features of this cartridge are as follows: (1) the solid-phase medium is a small silica monolithic disk; (2) it is sized to accommodate small sample volumes (150 µL of serum); and (3) it is suitable for processing multiple samples because all liquid permeation operations (e.g., degassing, washing, and elution) are performed through centrifugation. A blood drug level analysis method combining SPE and HPLC using a mass spectrometer or UV detector has been reported [16–28]. Our method is a combination of SPE and HPLC/UV. The SPE pretreatment involves almost the same procedure for each drug (Fig. 1), and the two mobile phases used in HPLC-UV are not changed for each drug to be measured. Therefore, this HPLC-UV platform allows simple analysis of blood levels of various drugs.

Second, we suggest that the use of a stable UV detector and an ODS silica monolithic column contributed to the success of this method. The periodic replacement of the guard column (about every 3 months) and monolithic column (every 6 months) ensured an extremely low possibility of column-related problems. There have been reports on the performance of TDM using monolithic columns [29–32]; however, in many studies, these columns were used for single drugs or classes of drugs. In this report, our HPLC-UV platform was effectively used to quantify patient serum levels of five different drugs (CBZ, PHT, LTG, VCM, and VRCZ) in our hospital. Our HPLC-UV platform could be applied to the in-hospital measurement of blood levels of more compounds, including the 15 drugs in previous research [11].

Third, the absolute calibration method using a one-point calibration line showed adequate performance for measuring blood concentrations in-hospital. Generally, the internal standard (IS) method is used for HPLC quantification, as accuracy is crucial. However, in our study, providing stable isotope labeling as IS for all drugs was considered impractical. Furthermore, the standard solution used to prepare the calibration curve must be easy to handle. In this study, quantitative validation was performed with one-point calibration curves, and no outliers were found for the values of accuracy, repeatability, and intermediate precision (as shown in Table S5). Meanwhile, standard reference material is needed for the preparation of a calibration curve to perform accuracy control with traceability according to certification standards in-hospital. Therefore, the preparation of standard solutions whose quality is guaranteed by the manufacturer is desirable.

The method discussed herein is expected to be used for in-hospital measurements that are currently outsourced. Many studies have used HPLC-MS/MS or HPLC-UV to analyze blood levels of VRCZ [33–39]. In addition, LTG has been analyzed using HPLC-UV [40–45], while mycophenolic acid and imatinib were measured using HPLC-UV [46, 47]. However, these methods are not scalable for measuring the blood levels of other drugs. Our HPLC-UV platform can be used to measure the blood levels of VRCZ and LTG, and can be extended to mycophenolic acid and imatinib [11], indicating that it is expected to show high versatility in the medical field.

The concept of this HPLC-UV platform is that the analysis can be performed in many hospitals and other facilities using relatively inexpensive equipment. Analytical costs are an important factor for TDM. Many Japanese hospitals are unable to measure blood levels of many drugs in-hospital because of measurement costs. Especially for hospitals that cannot sufficiently measure in-hospital drug blood levels, the introduction of this platform should increase the number of analyzed drugs and enable the expansion of TDM operations. Moreover, the health and economic benefits gained by TDM should also be considered. In recent years, TDM has been used in neuropsychopharmacology to increase the efficacy and safety of drug treatments and reduce healthcare costs [48]. Furthermore, it has been suggested that TDM dosing may be a cost-effective intervention in the administration of imatinib [49, 50]. As TDM operations expand through the use of our methods, we expect to see further evidence of its cost-effective benefits.

In recent years, the performance of regular TDM for seriously ill patients prescribed antibiotics (e.g., linezolid, teicoplanin, VCM, and VRCZ) has been recommended [51]. For the analysis of blood levels of such antimicrobial agents, HPLC-UV is reportedly suitable for facilities without expertise in LC-MS/MS [52]. In addition, there have been reports on the use of the HPLC-UV method for the analysis of blood concentrations of β-lactam antibiotics [9, 10, 53, 54]. This HPLC-UV platform is currently only applicable to VCM and VRCZ but is expected to be applied to other antimicrobial agents in the future.

We intend to further expand the range of applications of this HPLC-UV platform to contribute to TDM operations in hospitals and improved quality of medical care.

## Conclusion

This method can be performed without the need for special analytical techniques. Our HPLC-UV platform is expected to be used for measurement of blood levels of various drugs for in-hospital routine TDM.

### Electronic supplementary material

Below is the link to the electronic supplementary material.

Supplementary Material 1

## Data Availability

Data will be made available on request.

## Abbreviations

TDM: therapeutic drug monitoring
LBA: ligand-binding assay
HPLC: high-performance liquid chromatography
MS/MS: tandem mass spectrometry
UV: ultraviolet
CBZ: carbamazepine
PHT: phenytoin
LTG: lamotrigine
VRCZ: voriconazole
VCM: vancomycin
LOD: limit of detection
LLOQ: lower limit of quantitation
QC: quality control
RSD: relative standard deviation
