# Supplementary material for: Evaluation of the clinical and quantitative performance of a practical HPLC-UV platform for in-hospital routine therapeutic drug monitoring of multiple drugs
Source: J Pharm Health Care Sci. 2023 Oct 1;9:29. doi: 10.1186/s40780-023-00298-7 (PMC10544152; doi:10.1186/s40780-023-00298-7)
Supplement: Supplementary file 1 — Supplementary Material 1 [file 40780_2023_298_MOESM1_ESM.docx]

Supplementary data

# Evaluation of the clinical and quantitative performance of a practical HPLC-UV platform for in-hospital routine therapeutic drug monitoring of multiple drugs

Go Morikawa^1*^, Kazuto Fukami^2^, Yukiko Moriiwa^3^, Katsuko Okazawa^1^ and Akio Yanagida^3^

^1^ *Department of Pharmacy, Hokushin General Hospital, 1-5-63, Nishi, Nakano, Nagano 383-8505, Japan*

^2^ *Department of Clinical laboratory, Hokushin General Hospital, 1-5-63, Nishi, Nakano, Nagano 383-8505, Japan*

^3^ *Department of Biomedical Analysis, School of Pharmacy, Tokyo University of Pharmacy and Life Sciences, 1432-1 Horinouchi, Hachioji, Tokyo 192-0392, Japan*

^*^ Corresponding author. E-mail address: gomorikawa@hokushin-hosp.jp (G. Morikawa). Department of Pharmacy, Hokushin General Hospital, 1-5-63, Nishi, Nakano, Nagano 383-8505, Japan.

**Table S1 Optimized RP-HPLC conditions for drug quantification**.

| Drug ^a)^ | Mobile phase condition | | UV Detection wavelength  (nm) |
| --- | --- | --- | --- |
|  | Elution program & solvent composition ^b)^ | Flow rate  (mL/min) |  |
| CBZ | Isocratic elution of 40% A, for 3 min | 2.0 | 280 |
| PHT | Isocratic elution of 40% A, for 3 min | 2.0 | 220 |
| VCM | Linear gradient elution (See Table S2) | 2.0 | 235 |
| LTG | Isocratic elution of 25% A, for 3 min | 2.0 | 210 |
| VRCZ | Isocratic elution of 40% A, for 3 min | 2.0 | 254 |

^a)^ CBZ: carbamazepine, PHT: phenytoin, VCM: vancomycin, LTG: lamotrigine, VRCZ: voriconazole.

^b)^ Each mobile-phase solvent is a mixture of solvent A (CH_3_CN) and solvent B (10 mM acetate buffer, pH 5).

**Table S2. HPLC conditions for VCM.**

The mobile phase, consisting of solvent A (CH_3_CN) and solvent B (10 mM acetate buffer, pH 5), was delivered at 2.0 mL/min in gradient mode.

| Time (min) | %A | %B |
| --- | --- | --- |
| 0 | 5 | 95 |
| 3.0 | 30 | 70 |
| 3.1 | 60 | 40 |
| 4.5 | 60 | 40 |
| 4.6 | 5 | 95 |
| 7.0 | 5 | 95 |

**Table S3.** Recovery efficiency of the five drugs in the present quantification method using our HPLC-UV platform.

| Drug | | Recovery efficiency | |
| --- | --- | --- | --- |
| Name ^a)^ | Conc. in serum  (μg/mL) | Recovery rate ^b)^  (%) | SPE factor  *f* |
| CBZ ^c)^ | 20 | 96.6 | 1.04 |
| PHT ^c)^ | 20 | 87.9 | 1.14 |
| VCM | 20 | 85.2 | 1.17 |
| LTG ^c)^ | 20 | 94.3 | 1.06 |
| VRCZ ^c)^ | 5 | 108.5 | 0.92 |

^a)^ CBZ: carbamazepine, PHT: phenytoin, VCM: vancomycin, LTG: lamotrigine, VRCZ: voriconazole.

^b)^ Average value (n=4).

^c)^ This data was quoted from Ref. [11].

**Table S4.** Limit values of the five drugs in the present quantification method using our HPLC-UV platform.

| Drug | | Limit values | | |
| --- | --- | --- | --- | --- |
| Name ^a)^ | Conc. in serum  (μg/mL) | LOD ^b)^  (μg/mL) | LOQ ^c)^  (μg/mL) | LLOQ ^d)^  (μg/mL) |
| CBZ ^e)^ | 2 | 0.1 | 0.3 | 0.1 |
| PHT ^e)^ | 2 | 0.2 | 0.7 | 0.3 |
| VCM | 3 | 0.1 | 0.2 | 0.1 |
| LTG ^e)^ | 2 | 0.1 | 0.2 | 0.1 |
| VRCZ ^e)^ | 1 | 0.4 | 1.3 | 0.7 |

^a)^ CBZ: carbamazepine, PHT: phenytoin, VCM: vancomycin, LTG: lamotrigine, VRCZ: voriconazole.

^b)^ LOD: the limit of detection. ^c)^ LOQ: the limit of quantitation. ^d)^ LLOQ: the lower limit of quantitation.

^e)^ This data was quoted from Ref. [11].

**Table S5.** Accuracy and precision data of five drugs in the present quantification method using our HPLC-UV platform.

| Analyte ^a)^ | Conc. in serum (μg/mL) | | Accuracy ^b)^ (%) | | Repeatability ^c)^  (CV%) | | Intermediate precision ^d)^  (CV%) | |
| --- | --- | --- | --- | --- | --- | --- | --- | --- |
| CBZ ^e)^ | 2 |  | 98.6 |  | 6.0 |  | 4.5 |  |
|  | 10 |  | 102.3 |  | 1.5 |  | 2.7 |  |
|  | 20 |  | 100.0 |  | 3.0 |  | 3.0 |  |
|  |  |  |  |  |  |  |  |  |
| PHT ^e)^ | 2 |  | 100.2 |  | 7.7 |  | 4.1 |  |
|  | 10 |  | 102.8 |  | 5.9 |  | 1.4 |  |
|  | 20 |  | 100.0 |  | 2.3 |  | 3.7 |  |
|  |  |  |  |  |  |  |  |  |
| VCM | 1 |  | 105.7 |  | 10.6 |  | 18.6 |  |
|  | 10 |  | 91.5 |  | 8.2 |  | 9.2 |  |
|  | 50 |  | 91.3 |  | 5.1 |  | 8.7 |  |
|  |  |  |  |  |  |  |  |  |
| LTG ^e)^ | 2 |  | 95.8 |  | 3.1 |  | 4.3 |  |
|  | 10 |  | 102.1 |  | 3.3 |  | 2.5 |  |
|  | 20 |  | 104.7 |  | 5.4 |  | 3.0 |  |
|  |  |  |  |  |  |  |  |  |
| VRCZ ^e)^ | 1 |  | 91.9 |  | 1.7 |  | 2.2 |  |
|  | 2 |  | 90.0 |  | 1.1 |  | 1.4 |  |
|  | 5 |  | 95.2 |  | 2.1 |  | 1.6 |  |

^a)^ CBZ: carbamazepine, PHT: phenytoin, VCM: vancomycin, LTG: lamotrigine, VRCZ: voriconazole.

^b)^ The accuracy (%) was calculated from the data of five samples (n=5) at a given concentration of each drug.

^c)^ The repeatability (CV%) was calculated from quadruplicate samples (n=4).

^d)^ The intermediate precision (CV%) was calculated over a series of three analyses on different days (3 days).

^e)^ This data was quoted from Ref. [11].

**Table S6. Accuracy of the HPLC-UV platform in repeated drug measurements of patient sera.**

| No. | drugs | AVR (n=3) | | SD (n=3) | | RSD % | |
| --- | --- | --- | --- | --- | --- | --- | --- |
| 1 | carbamazepine | 5.9 |  | 0.2 |  | 3.4 |  |
| 2 | carbamazepine | 7.1 |  | 0.9 |  | 12.1 |  |
| 3 | carbamazepine | 8.0 |  | 0.1 |  | 1.2 |  |
| 4 | carbamazepine | 4.9 |  | 0.1 |  | 2.4 |  |
| 5 | carbamazepine | 7.2 |  | 0.1 |  | 1.1 |  |
| 6 | carbamazepine | 9.2 |  | 0.1 |  | 0.9 |  |
| 7 | carbamazepine | 9.2 |  | 0.2 |  | 2.4 |  |
| 8 | carbamazepine | 8.5 |  | 0.2 |  | 2.0 |  |
| 9 | carbamazepine | 7.6 |  | 0.4 |  | 5.4 |  |
| 10 | carbamazepine | 6.9 |  | 0.1 |  | 1.3 |  |
| 11 | carbamazepine | 6.6 |  | 0.2 |  | 3.2 |  |
| 12 | carbamazepine | 7.3 |  | 0.2 |  | 2.0 |  |
| 13 | carbamazepine | 7.5 |  | 0.2 |  | 2.1 |  |
| 14 | phenytoin | 13.7 |  | 0.3 |  | 2.1 |  |
| 15 | phenytoin | 6.4 |  | 0.2 |  | 2.6 |  |
| 16 | phenytoin | 10.0 |  | 0.1 |  | 1.4 |  |
| 17 | phenytoin | 11.9 |  | 1.5 |  | 12.2 |  |
| 18 | lamotrigine | 0.7 |  | 0.1 |  | 10.5 |  |
| 19 | lamotrigine | 5.8 |  | 1.0 |  | 16.7 |  |
| 20 | voriconazole | 0.6 |  | 0.0 |  | 1.1 |  |
| 21 | voriconazole | 4.1 |  | 0.3 |  | 8.3 |  |
